# Supplementary material for: A low-dimensional cognitive-network space in Alzheimer’s disease and frontotemporal dementia
Source: Alzheimers Res Ther. 2022 Dec 29;14:199. doi: 10.1186/s13195-022-01145-x (PMC9798659; doi:10.1186/s13195-022-01145-x)

**Methods**

**Relationship between cognitive components and cognitive networks**

Bootstrapping results were complemented through a stepwise-removal-of-data analysis. Spearman’s correlations between cognitive principal components (cogPC) scores and functional connectivity (FC) of each network were computed after removing one subject randomly at each step (*leave-and-take-one-out procedure*). In total, we performed 16 steps (corresponding to the exclusion of the 25% of the whole dataset for the last run of the procedure). This procedure was randomly repeated 10000 times to improve the robustness of the result, for a total of 160000 spearman’s correlation values computed for each network-connectivity coupling. The median p-values and Spearman’s coefficients of this convergent correlation-wise analysis distribution were then computed and plotted. We then assessed the statistical significance of median r values by means of a permutation approach. For each cogPC scores-network coupling we permuted the data 100 times (permutating FC values keeping fixed cogPC scores). At each permutation step we performed 100 random steps excluding until the 25% of the whole dataset, as in the convergent correlation-wise analysis. This procedure led to the same amount of Spearman’s (permuted) correlation values (n=160000). Median stepwise-removal r values were z-scored according to the mean and standard deviation of this resulting permutation distribution and the corresponding p-values were computed.

**Results**

**Brain functional networks and connectivity-cognitive coupling**

The stepwise-removal-of-data analysis confirmed the main convergent analysis. We reported a median rho correlation of 0.303 between the memory component and the DMN (p_median_ DMN=0.026), indicating that lower cognitive performance was associated with lower FC. Similarly, the emotion-language component showed a median rho correlation around 0.30 for both the DMN and VAN (p_median_ DMN=0.023; p_median_ VAN=0.043). These median correlation values compared to a permutation distribution showed statistical significance for the DMN (memory component: p=0.016; emotion-language component p=0.042) and near to the significance level for the VAN (emotion-language component p=0.056) (**Supplementary Figure S2**). These results were confirmed after excluding outliers according to the 1.5 interquartile range based on network distribution values (DMN-memory component: median rho correlation = 0.272; p_median_=0.044; DMN-emotion-language component: median rho correlation = 0.309; p_median_=0.015; VAN-emotion-language component: median rho correlation = 0.289; p_median_=0.014). By contrast, DAN and FPN showed no significant association with any component. Similarly, the visuo-spatial attentional component showed no significant correlation with the cognitive networks (**Supplementary Figure S2**).

**Figure Supplementary S1. Network differences between patients and controls.**

Different involvement in AD and bvFTD was reported compared to controls. AD showed lower connectivity in the dorsal-attention network, frontoparietal network and default mode network (top panel). Compared to controls, bvFTD showed reduced connectivity only in the ventral-attention network (bottom panel).

**
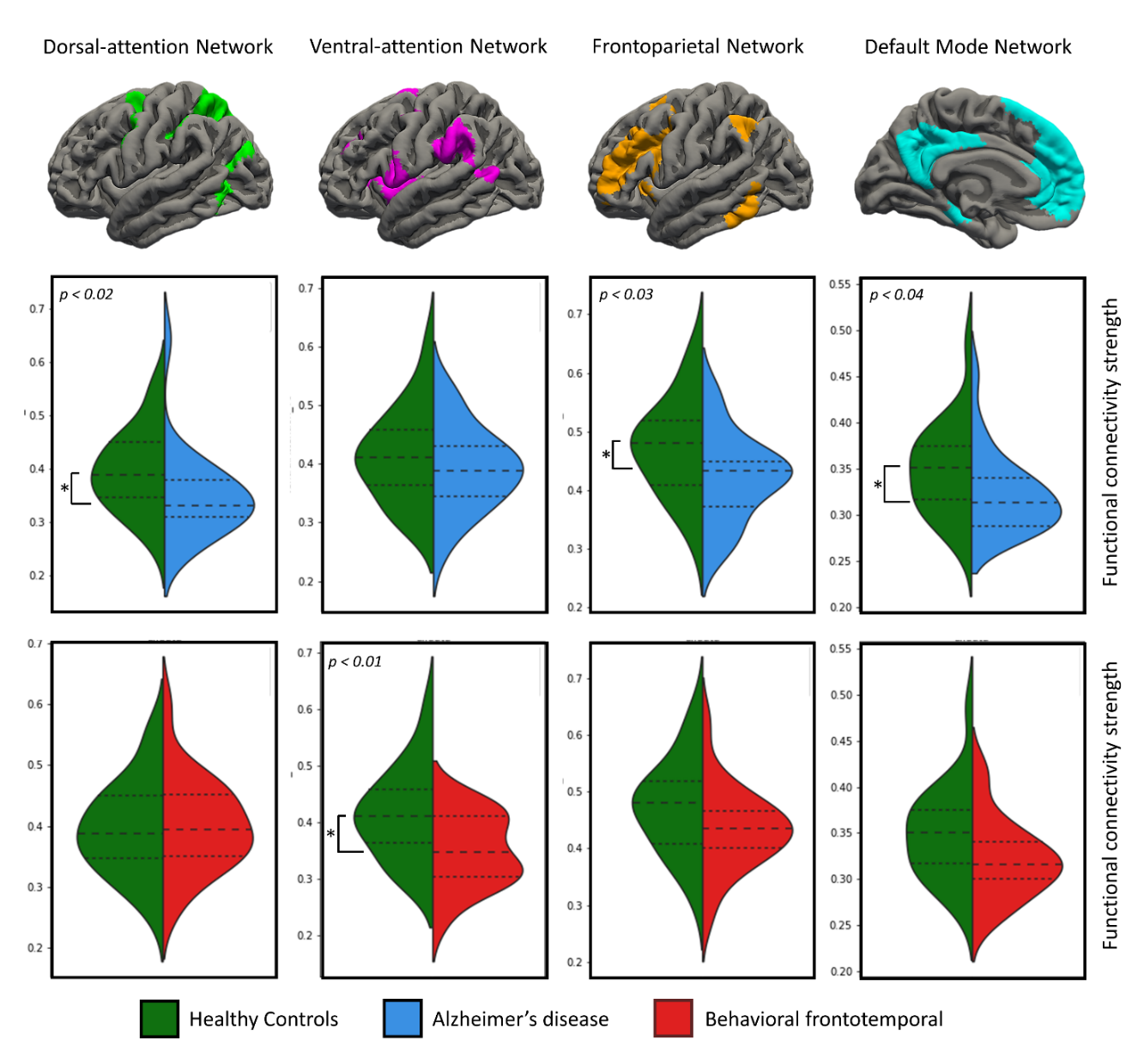
**

**Supplementary Figure S2. Stepwise-removal analysis**

Spearman’s correlation (blue distribution) and p-values (red distribution) for the stepwise-removal analysis in the whole dataset. Correlations were computed for each cognitive network (default mode network, ventral and dorsal attention network, and frontoparietal network) and cognitive component scores. Panel A: correlation values distribution between connectivity and cogPC1 (memory component); Panel B: correlation values distribution between connectivity and the cogPC2 (emotion-language component). Panel C: correlation values distribution between connectivity and the cogPC3 (visuo-spatial attentional component). Panel D: permutation distributions (in blue) and the significant convergent median p-values (red lines).

**
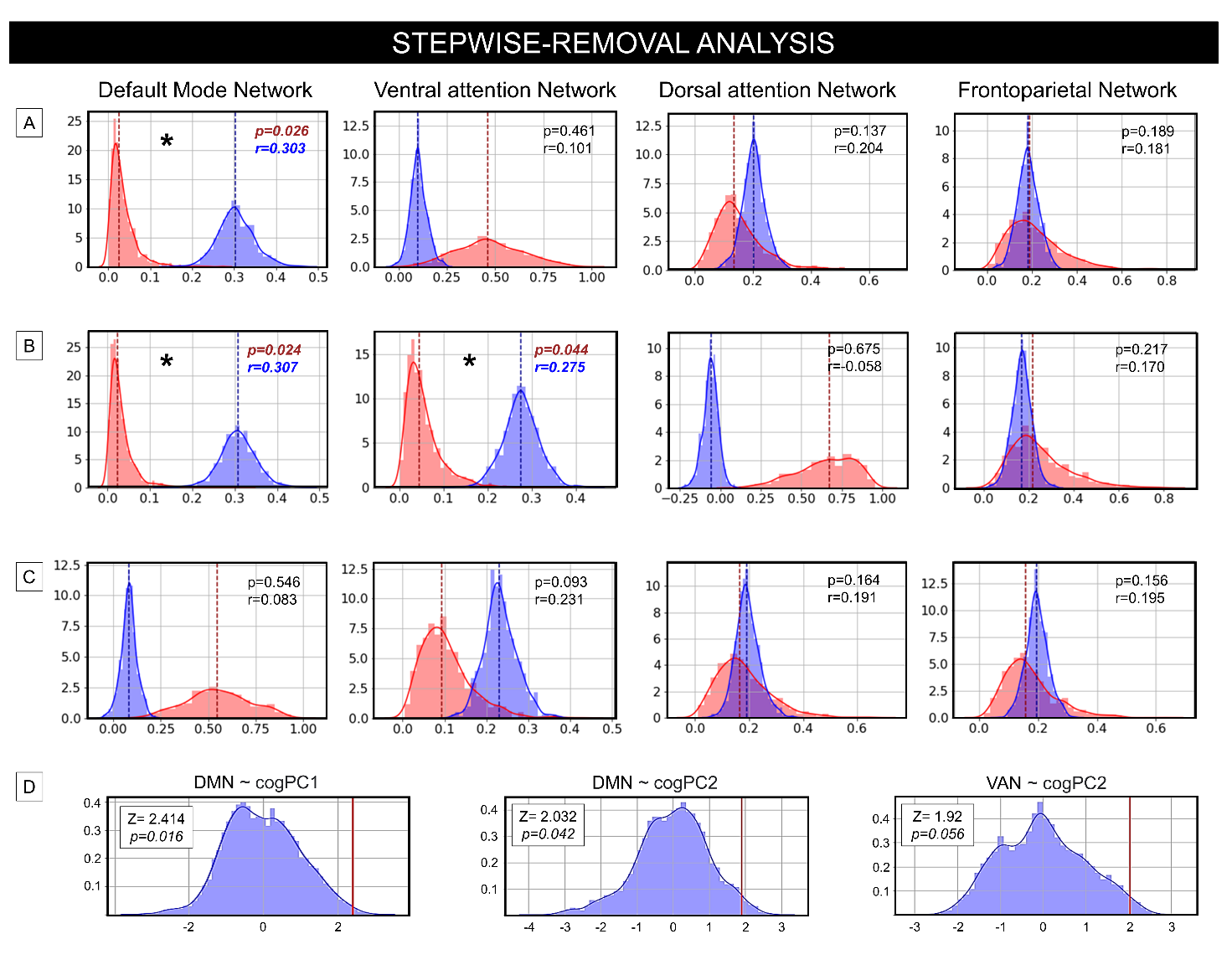
**

**Supplementary Figure S3. Relationship between connectivity and cognitive components.**

The main Interaction effect analysis between network, diagnosis and cognition was replicated in the dementia cohort, excluding healthy controls. Top panel: bar plots of the diagnosis*cognitive component significance interaction with the attentional networks. Bottom panel: scatter plot of the interaction for the non-memory components and the attentional networks.

**
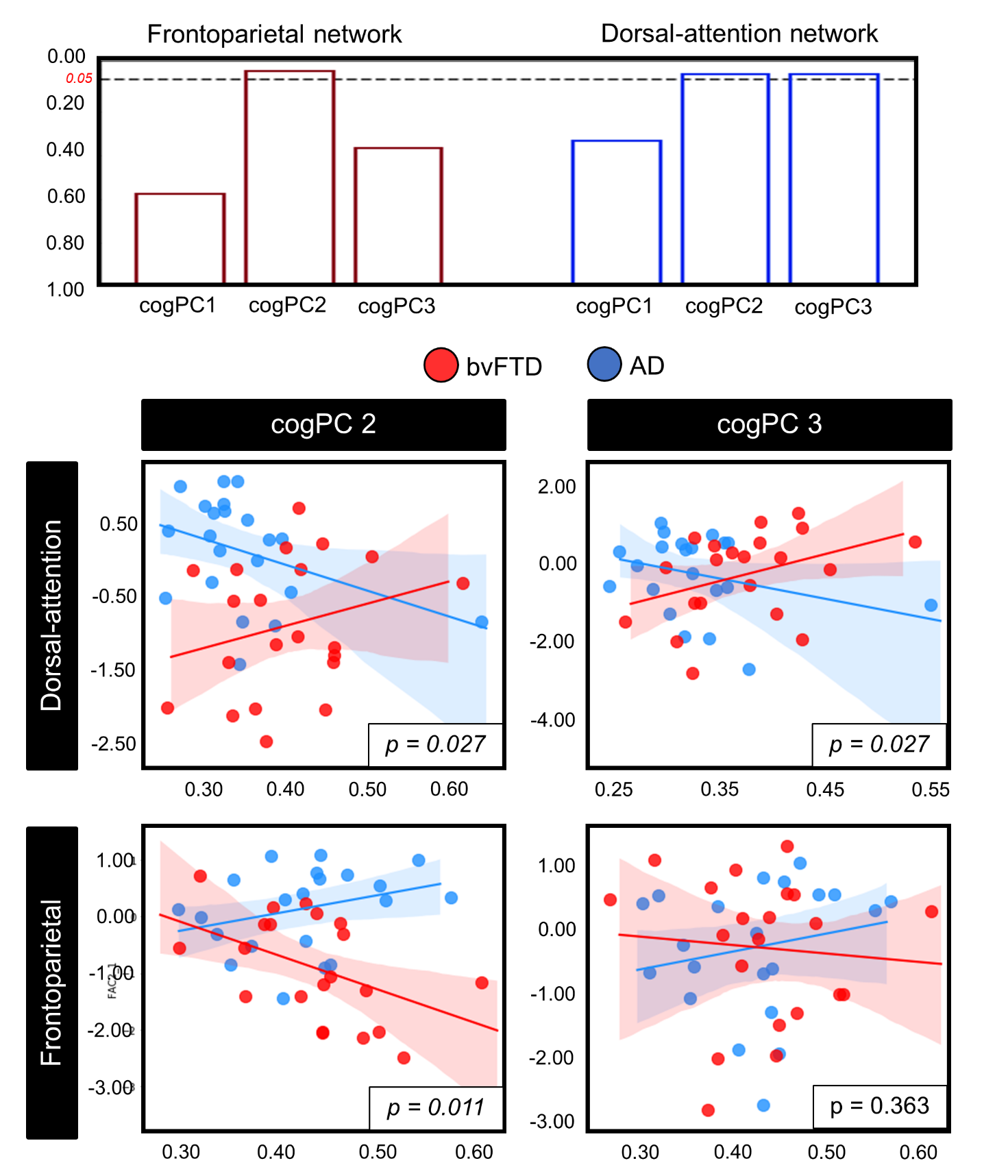
**

**Figure Supplementary S4. Within-diagnosis divergent cognitive-network coupling**

A within-diagnosis cognitive * networks (frontoparietal – FPN – and dorsal attention – DAN) analysis showed similar divergent network-cognitive coupling patterns in both Alzheimer’s disease (AD) and behavioral frontotemporal (bvFTD) patients. Significant divergent effects were reported between the emotion-language component (component 2) and the attentional networks.


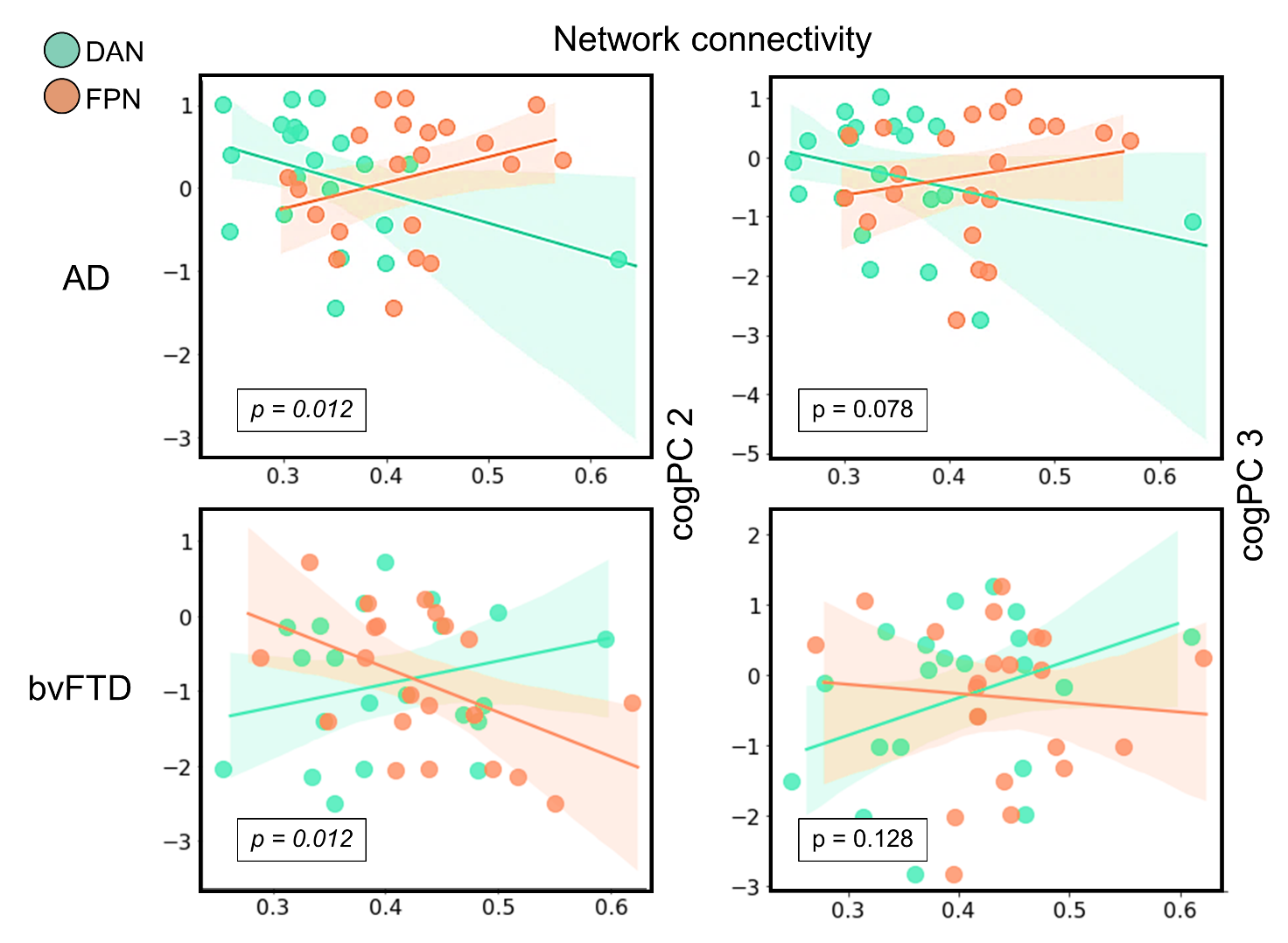

Supplement: Supplementary file 1 — Additional file 1. Supplementary material. [file 13195_2022_1145_MOESM1_ESM.docx]
